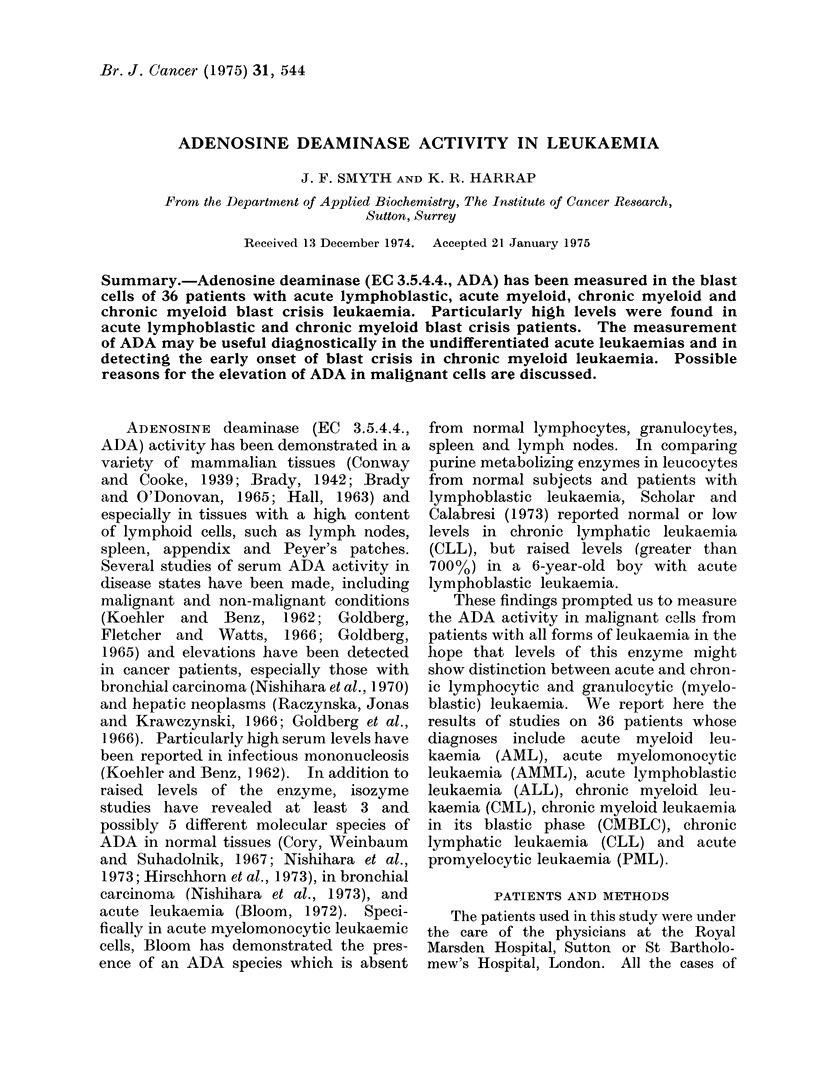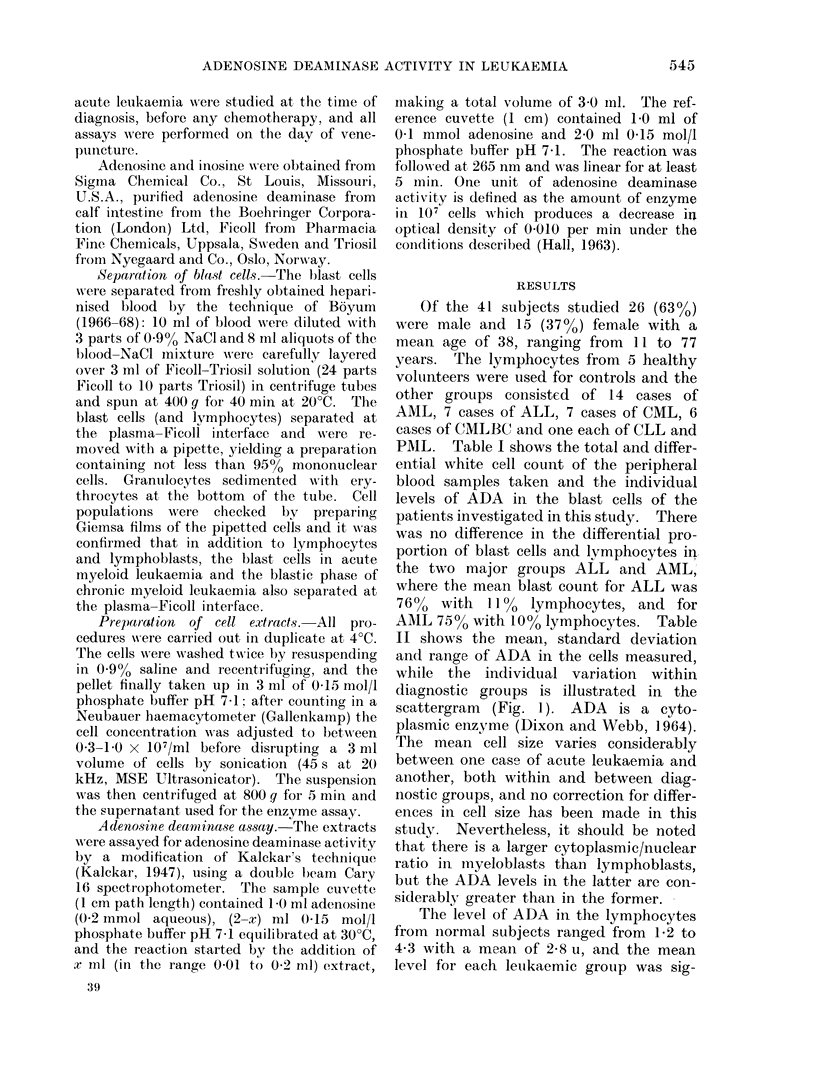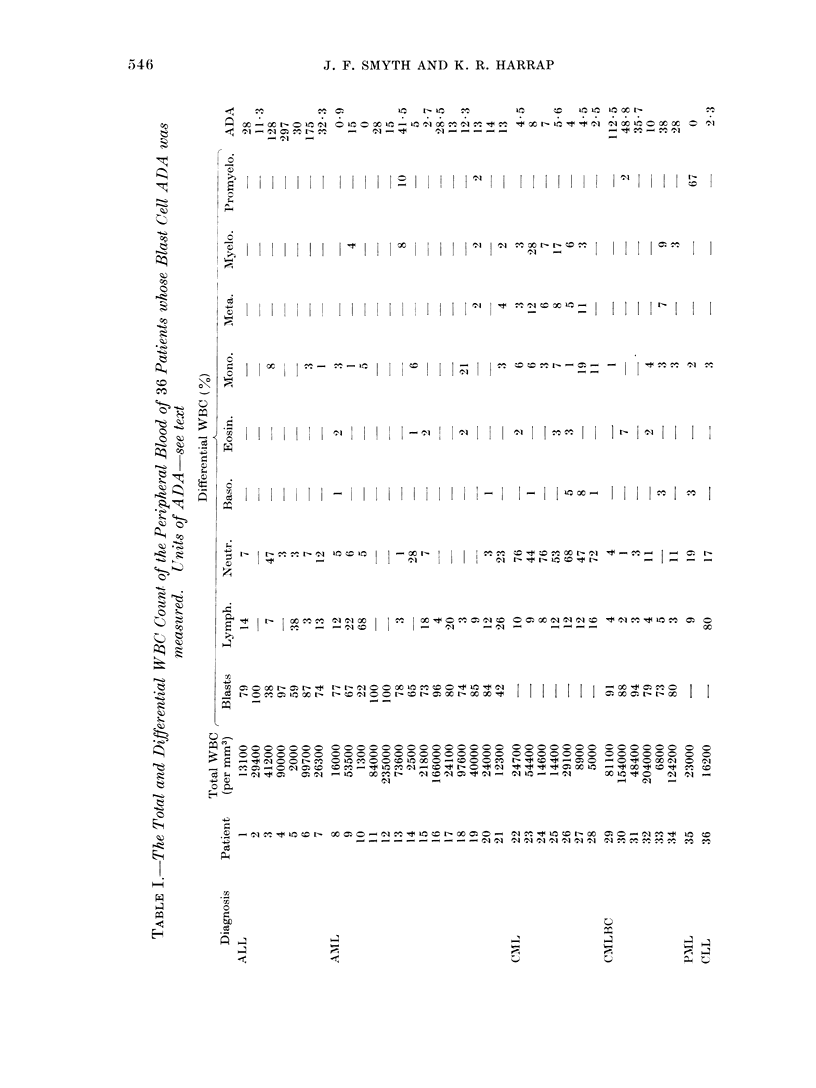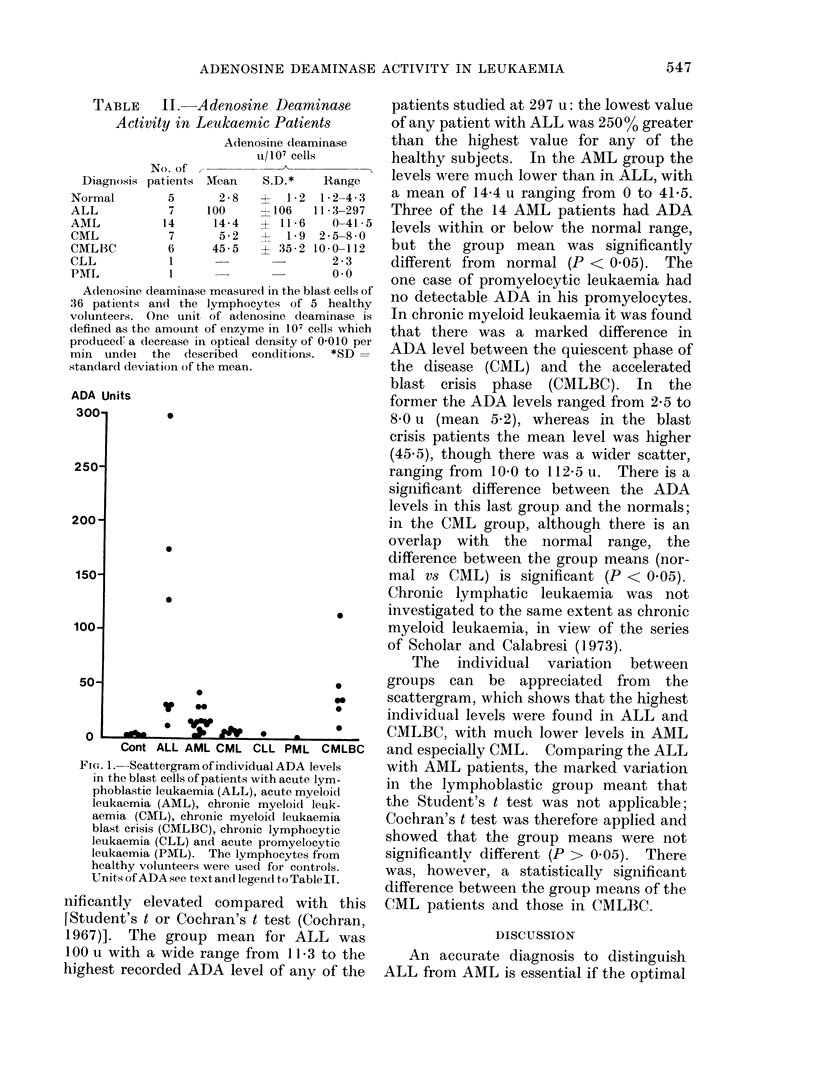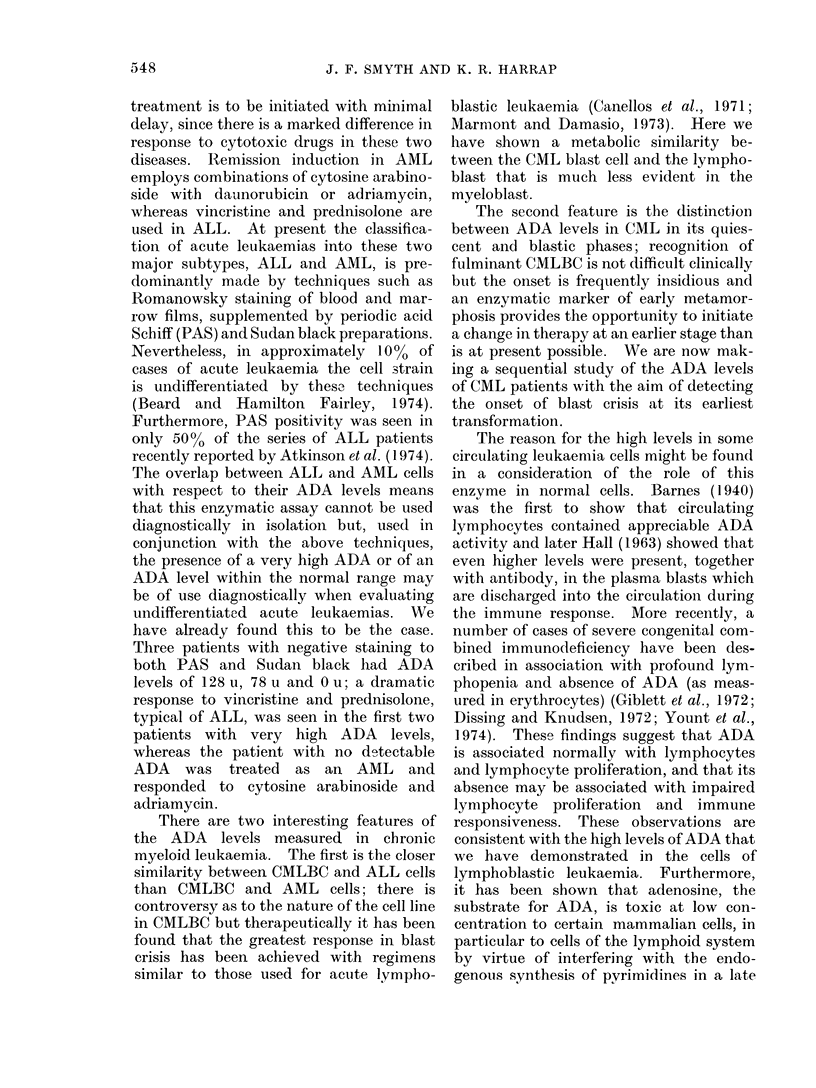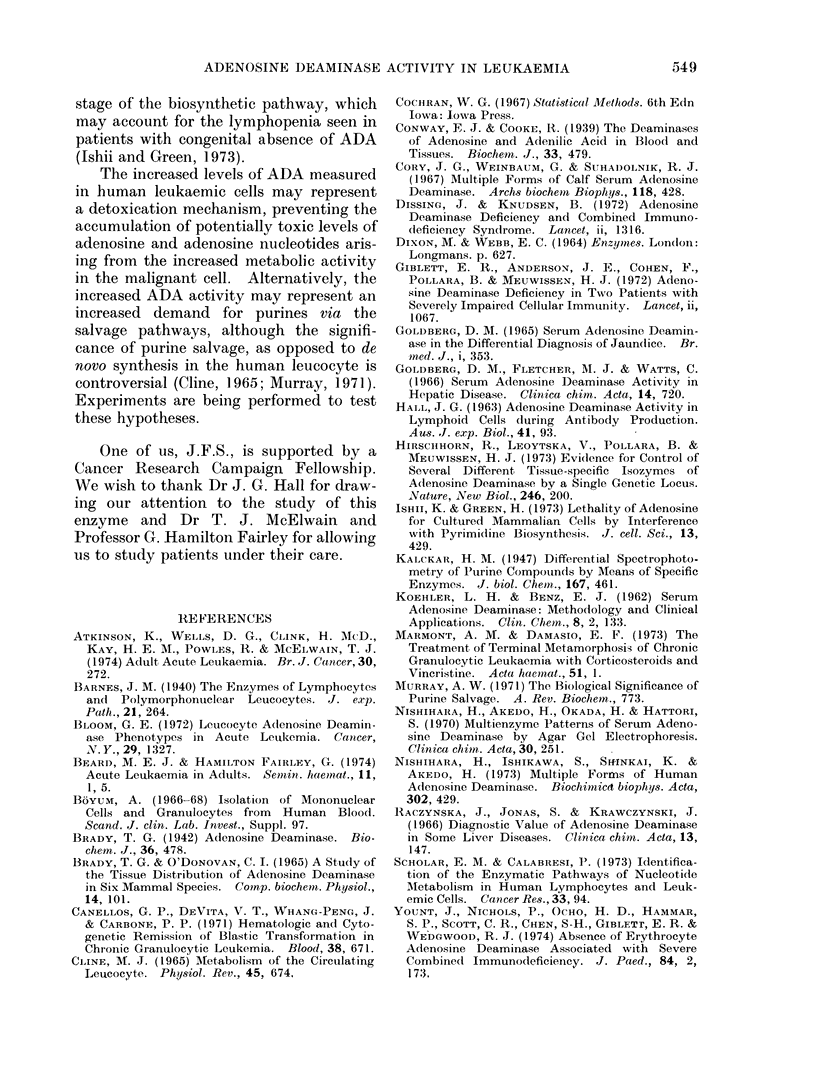# Adenosine deaminase activity in leukaemia.

**DOI:** 10.1038/bjc.1975.95

**Published:** 1975-05

**Authors:** J. F. Smyth, K. R. Harrap

## Abstract

Adenosine deaminase (EC 3.5.4.4., ADA) has been measured in the blast cells of 36 patients with acute lymphoblastic, acute myeloid, chronic myeloid and chronic myeloid blast crisis leukaemia. Particularly high levels were found in acute lymphoblastic and chronic myeloid blast crisis patients. The measurement of ADA may be useful diagnostically in the undifferentiated acute leukaemias and in detecting the early onset of blast crisis in chronic myeloid leukaemia. Possible reasons for the elevation of ADA in malignant cells are discussed.


*Br. J. Cancer* (1975) **31**, 544

### ADENOSINE DEAMINASE ACTIVITY IN LEUKAEMIA

J. F. SMYTH AND K. R. HARRAP

*From the Department of Applied Biochemistry, The Institute of Cancer Research, Sutton, Surrey*

Received 13 December 1974. Accepted 21 January 1975

**Summary.—Adenosine deaminase (EC 3.5.4.4., ADA) has been measured in the blast cells of 36 patients with acute lymphoblastic, acute myeloid, chronic myeloid and chronic myeloid blast crisis leukaemia. Particularly high levels were found in acute lymphoblastic and chronic myeloid blast crisis patients. The measurement of ADA may be useful diagnostically in the undifferentiated acute leukaemias and in detecting the early onset of blast crisis in chronic myeloid leukaemia. Possible reasons for the elevation of ADA in malignant cells are discussed.**

ADENOSINE deaminase (EC 3.5.4.4., ADA) activity has been demonstrated in a variety of mammalian tissues (Conway and Cooke, 1939; Brady, 1942; Brady and O'Donovan, 1965; Hall, 1963) and especially in tissues with a high content of lymphoid cells, such as lymph nodes, spleen, appendix and Peyer's patches. Several studies of serum ADA activity in disease states have been made, including malignant and non-malignant conditions (Koehler and Benz, 1962; Goldberg, Fletcher and Watts, 1966; Goldberg, 1965) and elevations have been detected in cancer patients, especially those with bronchial carcinoma (Nishihara *et al.*, 1970) and hepatic neoplasms (Raczynska, Jonas and Krawczynski, 1966; Goldberg *et al.*, 1966). Particularly high serum levels have been reported in infectious mononucleosis (Koehler and Benz, 1962). In addition to raised levels of the enzyme, isozyme studies have revealed at least 3 and possibly 5 different molecular species of ADA in normal tissues (Cory, Weinbaum and Suhadolnik, 1967; Nishihara *et al.*, 1973; Hirschhorn *et al.*, 1973), in bronchial carcinoma (Nishihara *et al.*, 1973), and acute leukaemia (Bloom, 1972). Specifically in acute myelomonocytic leukaemic cells, Bloom has demonstrated the presence of an ADA species which is absent from normal lymphocytes, granulocytes, spleen and lymph nodes. In comparing purine metabolizing enzymes in leucocytes from normal subjects and patients with lymphoblastic leukaemia, Scholar and Calabresi (1973) reported normal or low levels in chronic lymphatic leukaemia (CLL), but raised levels (greater than 700%) in a 6-year-old boy with acute lymphoblastic leukaemia.

These findings prompted us to measure the ADA activity in malignant cells from patients with all forms of leukaemia in the hope that levels of this enzyme might show distinction between acute and chronic lymphocytic and granulocytic (myeloblastic) leukaemia. We report here the results of studies on 36 patients whose diagnoses include acute myeloid leukaemia (AML), acute myelomonocytic leukaemia (AMML), acute lymphoblastic leukaemia (ALL), chronic myeloid leukaemia (CML), chronic myeloid leukaemia in its blastic phase (CMBLC), chronic lymphatic leukaemia (CLL) and acute promyelocytic leukaemia (PML).

### PATIENTS AND METHODS

The patients used in this study were under the care of the physicians at the Royal Marsden Hospital, Sutton or St Bartholomew's Hospital, London. All the cases of acute leukaemia were studied at the time of diagnosis, before any chemotherapy, and all assays were performed on the day of venepuncture.

Adenosine and inosine were obtained from Sigma Chemical Co., St Louis, Missouri, U.S.A., purified adenosine deaminase from calf intestine from the Boehringer Corporation (London) Ltd, Ficoll from Pharmacia Fine Chemicals, Uppsala, Sweden and Triosil from Nyegaard and Co., Oslo, Norway.

*Separation of blast cells.*—The blast cells were separated from freshly obtained heparinised blood by the technique of Böyum (1966–68): 10 ml of blood were diluted with 3 parts of 0·9% NaCl and 8 ml aliquots of the blood–NaCl mixture were carefully layered over 3 ml of Ficoll–Triosil solution (24 parts Ficoll to 10 parts Triosil) in centrifuge tubes and spun at 400 *g* for 40 min at 20°C. The blast cells (and lymphocytes) separated at the plasma–Ficoll interface and were removed with a pipette, yielding a preparation containing not less than 95% mononuclear cells. Granulocytes sedimented with erythrocytes at the bottom of the tube. Cell populations were checked by preparing Giemsa films of the pipetted cells and it was confirmed that in addition to lymphocytes and lymphoblasts, the blast cells in acute myeloid leukaemia and the blastic phase of chronic myeloid leukaemia also separated at the plasma–Ficoll interface.

*Preparation of cell extracts.*—All procedures were carried out in duplicate at 4°C. The cells were washed twice by resuspending in 0·9% saline and recentrifuging, and the pellet finally taken up in 3 ml of 0·15 mol/l phosphate buffer pH 7·1; after counting in a Neubauer haemacytometer (Gallenkamp) the cell concentration was adjusted to between $0{\cdot}3$–$1{\cdot}0 \times 10^7$/ml before disrupting a 3 ml volume of cells by sonication (45 s at 20 kHz, MSE Ultrasonicator). The suspension was then centrifuged at 800 *g* for 5 min and the supernatant used for the enzyme assay.

*Adenosine deaminase assay.*—The extracts were assayed for adenosine deaminase activity by a modification of Kalckar's technique (Kalckar, 1947), using a double beam Cary 16 spectrophotometer. The sample cuvette (1 cm path length) contained 1·0 ml adenosine (0·2 mmol aqueous), ($2-x$) ml 0·15 mol/l phosphate buffer pH 7·1 equilibrated at 30°C, and the reaction started by the addition of $x$ ml (in the range 0·01 to 0·2 ml) extract, making a total volume of 3·0 ml. The reference cuvette (1 cm) contained 1·0 ml of 0·1 mmol adenosine and 2·0 ml 0·15 mol/l phosphate buffer pH 7·1. The reaction was followed at 265 nm and was linear for at least 5 min. One unit of adenosine deaminase activity is defined as the amount of enzyme in $10^7$ cells which produces a decrease in optical density of 0·010 per min under the conditions described (Hall, 1963).

### RESULTS

Of the 41 subjects studied 26 (63%) were male and 15 (37%) female with a mean age of 38, ranging from 11 to 77 years. The lymphocytes from 5 healthy volunteers were used for controls and the other groups consisted of 14 cases of AML, 7 cases of ALL, 7 cases of CML, 6 cases of CMLBC and one each of CLL and PML. Table I shows the total and differential white cell count of the peripheral blood samples taken and the individual levels of ADA in the blast cells of the patients investigated in this study. There was no difference in the differential proportion of blast cells and lymphocytes in the two major groups ALL and AML, where the mean blast count for ALL was 76% with 11% lymphocytes, and for AML 75% with 10% lymphocytes. Table II shows the mean, standard deviation and range of ADA in the cells measured, while the individual variation within diagnostic groups is illustrated in the scattergram (Fig. 1). ADA is a cytoplasmic enzyme (Dixon and Webb, 1964). The mean cell size varies considerably between one case of acute leukaemia and another, both within and between diagnostic groups, and no correction for differences in cell size has been made in this study. Nevertheless, it should be noted that there is a larger cytoplasmic/nuclear ratio in myeloblasts than lymphoblasts, but the ADA levels in the latter are considerably greater than in the former.

The level of ADA in the lymphocytes from normal subjects ranged from 1·2 to 4·3 with a mean of 2·8 u, and the mean level for each leukaemic group was sig-

TABLE I.—*The Total and Differential WBC Count of the Peripheral Blood of* 36 *Patients whose Blast Cell ADA was measured. Units of ADA—see text*

| Diagnosis | Patient | Total WBC (per mm³) | Differential WBC (%) Blasts | Lymph. | Neutr. | Baso. | Eosin. | Mono. | Meta. | Myelo. | Promyelo. | ADA |
|---|---|---|---|---|---|---|---|---|---|---|---|---|
| ALL | 1 | 13100 | 79 | 14 | 7 | — | — | — | — | — | — | 28 |
| | 2 | 29400 | 100 | — | — | — | — | — | — | — | — | 11·3 |
| | 3 | 41200 | 38 | 7 | 47 | — | — | 8 | — | — | — | 128 |
| | 4 | 90000 | 97 | — | 3 | — | — | — | — | — | — | 297 |
| | 5 | 2000 | 59 | 38 | 3 | — | — | — | — | — | — | 30 |
| | 6 | 99700 | 87 | 3 | 7 | — | — | 3 | — | — | — | 175 |
| | 7 | 26300 | 74 | 13 | 12 | — | — | 1 | — | — | — | 32·3 |
| AML | 8 | 16000 | 77 | 12 | 5 | 1 | 2 | 3 | — | — | — | 0·9 |
| | 9 | 53500 | 67 | 22 | 6 | — | — | 1 | — | 4 | — | 15 |
| | 10 | 1300 | 22 | 68 | 5 | — | — | 5 | — | — | — | 0 |
| | 11 | 84000 | 100 | — | — | — | — | — | — | — | — | 28 |
| | 12 | 235000 | 100 | — | — | — | — | — | — | — | — | 15 |
| | 13 | 73600 | 78 | 3 | 1 | — | — | — | — | 8 | 10 | 41·5 |
| | 14 | 2500 | 65 | — | 28 | — | 1 | 6 | — | — | — | 5 |
| | 15 | 21800 | 73 | 18 | 7 | — | 2 | — | — | — | — | 2·7 |
| | 16 | 166000 | 96 | 4 | — | — | — | — | — | — | — | 28·5 |
| | 17 | 24100 | 80 | 20 | — | — | — | — | — | — | — | 13 |
| | 18 | 97600 | 74 | 3 | — | — | 2 | 21 | — | — | — | 12·3 |
| | 19 | 40000 | 85 | 9 | — | — | — | — | 2 | 2 | 2 | 13 |
| | 20 | 24000 | 84 | 12 | 3 | 1 | — | — | — | — | — | 14 |
| | 21 | 12300 | 42 | 26 | 23 | — | — | 3 | 4 | 2 | — | 13 |
| CML | 22 | 24700 | — | 10 | 76 | — | 2 | 6 | 3 | 3 | — | 4·5 |
| | 23 | 54400 | — | 9 | 44 | 1 | — | 6 | 12 | 28 | — | 8 |
| | 24 | 14600 | — | 8 | 76 | — | — | 3 | 6 | 7 | — | 7 |
| | 25 | 14400 | — | 12 | 53 | — | 3 | 7 | 8 | 17 | — | 5·6 |
| | 26 | 29100 | — | 12 | 68 | 5 | 3 | 1 | 5 | 6 | — | 4 |
| | 27 | 8900 | — | 12 | 47 | 8 | — | 19 | 11 | 3 | — | 4·5 |
| | 28 | 5000 | — | 16 | 72 | 1 | — | 11 | — | — | — | 2·5 |
| CMLBC | 29 | 81100 | 91 | 4 | 4 | — | — | 1 | — | — | — | 112·5 |
| | 30 | 154000 | 88 | 2 | 1 | — | 7 | — | — | — | 2 | 48·8 |
| | 31 | 48400 | 94 | 3 | 3 | — | — | — | — | — | — | 35·7 |
| | 32 | 204000 | 79 | 4 | 11 | — | 2 | 4 | — | — | — | 10 |
| | 33 | 6800 | 73 | 5 | — | 3 | — | 3 | 7 | 9 | — | 38 |
| | 34 | 124200 | 80 | 3 | 11 | — | — | 3 | — | 3 | — | 28 |
| PML | 35 | 23000 | — | 9 | 19 | 3 | — | 2 | — | — | 67 | 0 |
| CLL | 36 | 16200 | — | 80 | 17 | — | — | 3 | — | — | — | 2·3 |

TABLE II.—*Adenosine Deaminase Activity in Leukaemic Patients*

| Diagnosis | No. of patients | Adenosine deaminase u/$10^7$ cells Mean | S.D.* | Range |
|---|---|---|---|---|
| Normal | 5 | 2·8 | ± 1·2 | 1·2–4·3 |
| ALL | 7 | 100 | ±106 | 11·3–297 |
| AML | 14 | 14·4 | ± 11·6 | 0–41·5 |
| CML | 7 | 5·2 | ± 1·9 | 2·5–8·0 |
| CMLBC | 6 | 45·5 | ± 35·2 | 10·0–112 |
| CLL | 1 | — | — | 2·3 |
| PML | 1 | — | — | 0·0 |

Adenosine deaminase measured in the blast cells of 36 patients and the lymphocytes of 5 healthy volunteers. One unit of adenosine deaminase is defined as the amount of enzyme in $10^7$ cells which produced a decrease in optical density of 0·010 per min under the described conditions. *SD = standard deviation of the mean.

FIG. 1.—Scattergram of individual ADA levels in the blast cells of patients with acute lymphoblastic leukaemia (ALL), acute myeloid leukaemia (AML), chronic myeloid leukaemia (CML), chronic myeloid leukaemia blast crisis (CMLBC), chronic lymphocytic leukaemia (CLL) and acute promyelocytic leukaemia (PML). The lymphocytes from healthy volunteers were used for controls. Units of ADA see text and legend to Table II.

nificantly elevated compared with this [Student's *t* or Cochran's *t* test (Cochran, 1967)]. The group mean for ALL was 100 u with a wide range from 11·3 to the highest recorded ADA level of any of the patients studied at 297 u: the lowest value of any patient with ALL was 250% greater than the highest value for any of the healthy subjects. In the AML group the levels were much lower than in ALL, with a mean of 14·4 u ranging from 0 to 41·5. Three of the 14 AML patients had ADA levels within or below the normal range, but the group mean was significantly different from normal ($P < 0·05$). The one case of promyelocytic leukaemia had no detectable ADA in his promyelocytes. In chronic myeloid leukaemia it was found that there was a marked difference in ADA level between the quiescent phase of the disease (CML) and the accelerated blast crisis phase (CMLBC). In the former the ADA levels ranged from 2·5 to 8·0 u (mean 5·2), whereas in the blast crisis patients the mean level was higher (45·5), though there was a wider scatter, ranging from 10·0 to 112·5 u. There is a significant difference between the ADA levels in this last group and the normals; in the CML group, although there is an overlap with the normal range, the difference between the group means (normal *vs* CML) is significant ($P < 0·05$). Chronic lymphatic leukaemia was not investigated to the same extent as chronic myeloid leukaemia, in view of the series of Scholar and Calabresi (1973).

The individual variation between groups can be appreciated from the scattergram, which shows that the highest individual levels were found in ALL and CMLBC, with much lower levels in AML and especially CML. Comparing the ALL with AML patients, the marked variation in the lymphoblastic group meant that the Student's *t* test was not applicable; Cochran's *t* test was therefore applied and showed that the group means were not significantly different ($P > 0·05$). There was, however, a statistically significant difference between the group means of the CML patients and those in CMLBC.

### DISCUSSION

An accurate diagnosis to distinguish ALL from AML is essential if the optimal treatment is to be initiated with minimal delay, since there is a marked difference in response to cytotoxic drugs in these two diseases. Remission induction in AML employs combinations of cytosine arabinoside with daunorubicin or adriamycin, whereas vincristine and prednisolone are used in ALL. At present the classification of acute leukaemias into these two major subtypes, ALL and AML, is predominantly made by techniques such as Romanowsky staining of blood and marrow films, supplemented by periodic acid Schiff (PAS) and Sudan black preparations. Nevertheless, in approximately 10% of cases of acute leukaemia the cell strain is undifferentiated by these techniques (Beard and Hamilton Fairley, 1974). Furthermore, PAS positivity was seen in only 50% of the series of ALL patients recently reported by Atkinson *et al.* (1974). The overlap between ALL and AML cells with respect to their ADA levels means that this enzymatic assay cannot be used diagnostically in isolation but, used in conjunction with the above techniques, the presence of a very high ADA or of an ADA level within the normal range may be of use diagnostically when evaluating undifferentiated acute leukaemias. We have already found this to be the case. Three patients with negative staining to both PAS and Sudan black had ADA levels of 128 u, 78 u and 0 u; a dramatic response to vincristine and prednisolone, typical of ALL, was seen in the first two patients with very high ADA levels, whereas the patient with no detectable ADA was treated as an AML and responded to cytosine arabinoside and adriamycin.

There are two interesting features of the ADA levels measured in chronic myeloid leukaemia. The first is the closer similarity between CMLBC and ALL cells than CMLBC and AML cells; there is controversy as to the nature of the cell line in CMLBC but therapeutically it has been found that the greatest response in blast crisis has been achieved with regimens similar to those used for acute lymphoblastic leukaemia (Canellos *et al.*, 1971; Marmont and Damasio, 1973). Here we have shown a metabolic similarity between the CML blast cell and the lymphoblast that is much less evident in the myeloblast.

The second feature is the distinction between ADA levels in CML in its quiescent and blastic phases; recognition of fulminant CMLBC is not difficult clinically but the onset is frequently insidious and an enzymatic marker of early metamorphosis provides the opportunity to initiate a change in therapy at an earlier stage than is at present possible. We are now making a sequential study of the ADA levels of CML patients with the aim of detecting the onset of blast crisis at its earliest transformation.

The reason for the high levels in some circulating leukaemia cells might be found in a consideration of the role of this enzyme in normal cells. Barnes (1940) was the first to show that circulating lymphocytes contained appreciable ADA activity and later Hall (1963) showed that even higher levels were present, together with antibody, in the plasma blasts which are discharged into the circulation during the immune response. More recently, a number of cases of severe congenital combined immunodeficiency have been described in association with profound lymphopenia and absence of ADA (as measured in erythrocytes) (Giblett *et al.*, 1972; Dissing and Knudsen, 1972; Yount *et al.*, 1974). These findings suggest that ADA is associated normally with lymphocytes and lymphocyte proliferation, and that its absence may be associated with impaired lymphocyte proliferation and immune responsiveness. These observations are consistent with the high levels of ADA that we have demonstrated in the cells of lymphoblastic leukaemia. Furthermore, it has been shown that adenosine, the substrate for ADA, is toxic at low concentration to certain mammalian cells, in particular to cells of the lymphoid system by virtue of interfering with the endogenous synthesis of pyrimidines in a late stage of the biosynthetic pathway, which may account for the lymphopenia seen in patients with congenital absence of ADA (Ishii and Green, 1973).

The increased levels of ADA measured in human leukaemic cells may represent a detoxication mechanism, preventing the accumulation of potentially toxic levels of adenosine and adenosine nucleotides arising from the increased metabolic activity in the malignant cell. Alternatively, the increased ADA activity may represent an increased demand for purines *via* the salvage pathways, although the significance of purine salvage, as opposed to *de novo* synthesis in the human leucocyte is controversial (Cline, 1965; Murray, 1971). Experiments are being performed to test these hypotheses.

One of us, J.F.S., is supported by a Cancer Research Campaign Fellowship. We wish to thank Dr J. G. Hall for drawing our attention to the study of this enzyme and Dr T. J. McElwain and Professor G. Hamilton Fairley for allowing us to study patients under their care.

### REFERENCES

ATKINSON, K., WELLS, D. G., CLINK, H. McD., KAY, H. E. M., POWLES, R. & McELWAIN, T. J. (1974) Adult Acute Leukaemia. *Br. J. Cancer*, **30**, 272.

BARNES, J. M. (1940) The Enzymes of Lymphocytes and Polymorphonuclear Leucocytes. *J. exp. Path.*, **21**, 264.

BLOOM, G. E. (1972) Leucocyte Adenosine Deaminase Phenotypes in Acute Leukemia. *Cancer, N.Y.*, **29**, 1327.

BEARD, M. E. J. & HAMILTON FAIRLEY, G. (1974) Acute Leukaemia in Adults. *Semin. haemat.*, **11**, 1, 5.

BÖYUM, A. (1966–68) Isolation of Mononuclear Cells and Granulocytes from Human Blood. *Scand. J. clin. Lab. Invest.*, Suppl. 97.

BRADY, T. G. (1942) Adenosine Deaminase. *Biochem. J.*, **36**, 478.

BRADY, T. G. & O'DONOVAN, C. I. (1965) A Study of the Tissue Distribution of Adenosine Deaminase in Six Mammal Species. *Comp. biochem. Physiol.*, **14**, 101.

CANELLOS, G. P., DEVITA, V. T., WHANG-PENG, J. & CARBONE, P. P. (1971) Hematologic and Cytogenetic Remission of Blastic Transformation in Chronic Granulocytic Leukemia. *Blood*, **38**, 671.

CLINE, M. J. (1965) Metabolism of the Circulating Leucocyte. *Physiol. Rev.*, **45**, 674.

COCHRAN, W. G. (1967) *Statistical Methods.* 6th Edn Iowa: Iowa Press.

CONWAY, E. J. & COOKE, R. (1939) The Deaminases of Adenosine and Adenilic Acid in Blood and Tissues. *Biochem. J.*, **33**, 479.

CORY, J. G., WEINBAUM, G. & SUHADOLNIK, R. J. (1967) Multiple Forms of Calf Serum Adenosine Deaminase. *Archs biochem Biophys.*, **118**, 428.

DISSING, J. & KNUDSEN, B. (1972) Adenosine Deaminase Deficiency and Combined Immunodeficiency Syndrome. *Lancet*, ii, 1316.

DIXON, M. & WEBB, E. C. (1964) *Enzymes.* London: Longmans. p. 627.

GIBLETT, E. R., ANDERSON, J. E., COHEN, F., POLLARA, B. & MEUWISSEN, H. J. (1972) Adenosine Deaminase Deficiency in Two Patients with Severely Impaired Cellular Immunity. *Lancet*, ii, 1067.

GOLDBERG, D. M. (1965) Serum Adenosine Deaminase in the Differential Diagnosis of Jaundice. *Br. med. J.*, i, 353.

GOLDBERG, D. M., FLETCHER, M. J. & WATTS, C. (1966) Serum Adenosine Deaminase Activity in Hepatic Disease. *Clinica chim. Acta*, **14**, 720.

HALL, J. G. (1963) Adenosine Deaminase Activity in Lymphoid Cells during Antibody Production. *Aus. J. exp. Biol.*, **41**, 93.

HIRSCHHORN, R., LEOYTSKA, V., POLLARA, B. & MEUWISSEN, H. J. (1973) Evidence for Control of Several Different Tissue-specific Isozymes of Adenosine Deaminase by a Single Genetic Locus. *Nature, New Biol.*, **246**, 200.

ISHII, K. & GREEN, H. (1973) Lethality of Adenosine for Cultured Mammalian Cells by Interference with Pyrimidine Biosynthesis. *J. cell. Sci.*, **13**, 429.

KALCKAR, H. M. (1947) Differential Spectrophotometry of Purine Compounds by Means of Specific Enzymes. *J. biol. Chem.*, **167**, 461.

KOEHLER, L. H. & BENZ, E. J. (1962) Serum Adenosine Deaminase: Methodology and Clinical Applications. *Clin. Chem.*, **8**, 2, 133.

MARMONT, A. M. & DAMASIO, E. F. (1973) The Treatment of Terminal Metamorphosis of Chronic Granulocytic Leukaemia with Corticosteroids and Vincristine. *Acta haemat.*, **51**, 1.

MURRAY, A. W. (1971) The Biological Significance of Purine Salvage. *A. Rev. Biochem.*, 773.

NISHIHARA, H., AKEDO, H., OKADA, H. & HATTORI, S. (1970) Multienzyme Patterns of Serum Adenosine Deaminase by Agar Gel Electrophoresis. *Clinica chim. Acta*, **30**, 251.

NISHIHARA, H., ISHIKAWA, S., SHINKAI, K. & AKEDO, H. (1973) Multiple Forms of Human Adenosine Deaminase. *Biochimica biophys. Acta*, **302**, 429.

RACZYNSKA, J., JONAS, S. & KRAWCZYNSKI, J. (1966) Diagnostic Value of Adenosine Deaminase in Some Liver Diseases. *Clinica chim. Acta*, **13**, 147.

SCHOLAR, E. M. & CALABRESI, P. (1973) Identification of the Enzymatic Pathways of Nucleotide Metabolism in Human Lymphocytes and Leukemic Cells. *Cancer Res.*, **33**, 94.

YOUNT, J., NICHOLS, P., OCHO, H. D., HAMMAR, S. P., SCOTT, C. R., CHEN, S-H., GIBLETT, E. R. & WEDGWOOD, R. J. (1974) Absence of Erythrocyte Adenosine Deaminase Associated with Severe Combined Immunodeficiency. *J. Paed.*, **84**, 2, 173.